# Identification and Characterization of the Corazonin Receptor and Possible Physiological Roles of the Corazonin-Signaling Pathway in *Rhodnius prolixus*

**DOI:** 10.3389/fnins.2016.00357

**Published:** 2016-08-03

**Authors:** Zina Hamoudi, Angela B. Lange, Ian Orchard

**Affiliations:** Department of Biology, University of Toronto MississaugaMississauga, ON, Canada

**Keywords:** insect, peptide, corazonin, GPCR, dsRNA, heartbeat, ecdysis, cuticle color

## Abstract

Neuropeptides control many physiological and endocrinological processes in animals, acting as neuroactive chemicals within the central and peripheral nervous systems. Corazonin (CRZ) is one such neuropeptide that has a variety of physiological roles associated with control of heartbeat, ecdysis behavior initiation, and cuticle coloration. These physiological effects are mediated by the CRZ receptor (CRZR). In order to understand the role of the CRZ-signaling pathway in *Rhodnius prolixus*, the cDNA sequence encoding the Rhopr-CRZR was isolated and cloned revealing two splice variants (Rhopr-CRZR-α and β). Sequence analysis revealed characteristics of rhodopsin-like GPCRs. Rhopr-CRZR-α and β were dose-dependently activated by Rhopr-CRZ with EC_50_ values of 2.7 and 1 nM, respectively, when tested in a functional receptor assay using CHOKI-aeq cells. Neither receptors were activated by the evolutionarily-related peptides, Rhopr-AKH, or Rhopr-ACP. For 5th instars, qPCR revealed expression of Rhopr-CRZR transcript in the CNS, the dorsal vessel, abdominal dorsal epidermis, and prothoracic glands with associated fat body. Interestingly, transcript expression was also found in the female and male reproductive tissues. Rhopr-CRZR transcript was reduced after injection of dsCRZR into adult *R. prolixus*. In these insects, the basal heartbeat rate was reduced *in vivo*, and the increase in heartbeat frequency normally produced by CRZ on dorsal vessel *in vitro* was much reduced. No effect of dsCRZR injection was seen on ecdysis or coloration of the cuticle.

## Introduction

Fundamental questions in physiology revolve around understanding the means by which the nervous system communicates information (messages) throughout the organism. To do this, neurons use a variety of chemical messengers that act as neurotransmitters, neuromodulators, and neurohormones to allow for flexibility in the privacy, speed, and length of the message (Orchard et al., 2001). These chemical messengers often transduce their message via receptors that are located in the membrane on the target cell. The use of model organisms to study these signaling pathways is advantageous particularly since the genomes of a variety of organisms have been sequenced, allowing for the use of molecular tools to examine these pathways and their messengers; the most diverse messengers being via neuropeptides. These neuropeptides are often associated with distinct behaviors, although piecing together the overall integration of such behaviors is a challenge in neurobiology (Orchard et al., 2001).

The corazonin (CRZ)-signaling pathway is one such neuropeptide system that appears to have diverse functions throughout a variety of insect species despite CRZ having a conserved sequence (Veenstra, 2009). [Arg^7^]-CRZ, with the sequence pQTFQYSRGWTNamide, is the most abundant CRZ sequence present in insects (Veenstra, 1989, 1991; Hua et al., 2000). It has been proposed that this signaling pathway arose during protostome evolution alongside two other insect signaling pathways, the adipokinetic hormone (AKH), and the AKH/CRZ-related peptide (ACP) pathways (Hauser and Grimmelikhuijzen, 2014; Roch et al., 2014). Utilizing genomic data and using an *in silico* approach, Hauser and Grimmelikhuijzen (2014) proposed that an ancestral gonadotropin releasing hormone (GnRH)-like peptide and its receptor duplicated and diverged before the emergence of Mollusca and Annelida leading to an AKH-like peptide and receptor, as well as a CRZ-like peptide and receptor. Before the emergence of the Arthropoda, the AKH-signaling system duplicated leading to AKH and ACP systems along with the CRZ-signaling system (Roch et al., 2014).

CRZ increases the heartbeat rate in the cockroach *Periplaneta americana* (Veenstra, 1989), and recently was shown to do the same in *R. prolixus* (Patel et al., 2014). A study on *Anopheles gambiae*, however, showed that when CRZ and its receptor were knocked down by RNA interference (RNAi), there was no significant effect on heartbeat rate (Hillyer et al., 2012). In *Manduca sexta*, CRZ was found to initiate ecdysis behavior (Kim et al., 2004); however, this effect has not been reported in other insects. In locusts CRZ was found to induce dark body coloration (Tanaka, 1993; Tanaka and Pener, 1994; Tanaka and Yagi, 1997; Tawfik et al., 1999). When light colored solitarious nymphs were injected with [His^7^]-CRZ, *Schistocerca gregaria* (Tawfik et al., 1999), and *Locusta migratoria* (Tanaka, 2000) both developed black patterns; however, again CRZ did not have such an effect when tested in other insects such as *Galleria mellonella, Gryllus bimaculatus*, or *Bombyx. mori* (Hua et al., 2000; Hansen et al., 2010).

The physiological functions of CRZ are mediated by a signal-transducing membrane receptor, a G protein-coupled receptor (GPCR). All GPCRs possess a similar topographical structure that has been well-conserved through evolution (Caers et al., 2012). They all have seven transmembrane spanning helices each consisting of 20–30 amino acids, connected by three intracellular loops, and three extracellular loops. Moreover, they have an extracellular N-terminus, which holds several glycosylation sites, and an intracellular C-terminus with potential phosphorylation sites. The ligand binds to the extracellular part of the receptor, activating it and eliciting an intracellular response. Based on phylogenetic analysis, GPCRs can be classified into at least five subfamilies: rhodopsin, secretin, glutamate, adhesion and frizzled-tastes-2 (Fredriksson et al., 2003). The CRZ receptor (CRZR) belongs to the family of rhodopsin-like receptors, the largest subfamily of GPCRs. They are characterized by having a DRY motif at the border of the cytoplasmic end of the third transmembrane domain and a NSxxNPxxY domain in transmembrane seven (Oldham and Hamm, 2008). These motifs are believed to be important for G protein activation and/or protein stabilization (Ballesteros et al., 2001). To date, the CRZR has been cloned and characterized in the following insects: *Drosophila melanogaster* (Cazzamali et al., 2002; Park et al., 2002), *M. sexta* (Kim et al., 2004), *A. gambiae* (Belmont et al., 2006), and *B. mori* (Yang et al., 2013). The CRZR has also been cloned in *Musca domestica* (Sha et al., 2012).

*Rhodnius prolixus*, the vector of Chagas disease, is an ideal model for studying the control of physiological processes since many of these processes are driven by gorging on a blood meal once in each instar. This blood gorging then stimulates short-term physiological changes associated with digestion, and the elimination of excess salt and water, and long-term changes associated with growth and development. In the adult, blood gorging stimulates reproductive activity. Thus, these events can be initiated by a blood meal and accurately timed. In addition, the genome of *R. prolixus* was recently sequenced (Mesquita et al., 2015) enabling further analysis of signaling pathways associated with physiological and endocrinological processes. This paper examines the CRZ-signaling pathway in *R. prolixus*, cloning and de-orphaning the receptor and then manipulating its expression to gain insight into the physiological relevance of CRZ in *R. prolixus*.

## Materials and methods

### Animals

Fifth instar and adult *R. prolixus* were maintained in incubators at 60% humidity and 25°C. They were routinely fed once at each instar stage on defibrinated rabbits' blood (Hemostat Laboratories, Dixon, CA, USA; supplied by Cedarlane Laboratories Inc., Burlington, ON, Canada).

### Isolating and cloning the cDNA sequence encoding Rhopr-CRZR

Supercontig sequences representing the *R. prolixus* genome were imported to Geneious 4.7.6 to perform a local tBLASTn search with *D. melanogaster* CRZR (JC7896) amino acid sequence to mine for the CRZR in *R. prolixus*. Gene-specific primers, CRZR-FOR1 and CRZR-REV1 (Supplementary Table 1) were designed and used to amplify the partial cDNA sequence encoding Rhopr-CRZR using *R. prolixus* CNS cDNA as a template. The PCR reaction was performed with an S1000 thermal cycler (Bio-Rad Laboratories, Mississauga, ON, Canada) using the following temperature-cycling profiles: initial denaturation at 94°C for 3 min, followed by 39 cycles of 94°C for 30 s, 60°C for 30 s, and 72°C for 1 min, followed by a final extension at 72°C for 10 min. The PCR product was then column purified using the Axygen™ Axyprep™ PCR Clean-up Kit (Fisher Scientific Ltd., Ottawa, ON, Canada). The purified product was then cloned using pGEM-T Easy Vector (Promega, Madison, WI, USA). Positive clones containing the desired inserts were purified from an overnight culture using the PureLink® Quick Plasmid Miniprep Kit (Life Technologies, Carlsbad, CA, USA) and sent for sequencing at the Centre of Applied Genomics at the Hospital for Sick Children (MaRS Centre, Toronto, Ontario, Canada).

The complete 3′ end of Rhopr-CRZR cDNA was obtained using a modified 3′ rapid amplification of cDNA ends (RACE) PCR approach using fifth-instar *R. prolixus* CNS cDNA library (Paluzzi et al., 2008). Gene specific primers were designed using the open reading frame (ORF) and used in combination with plasmid-specific primers (Supplementary Table 1). A series of nested PCRs was performed in succession using three gene-specific forward primers (CRZR-RACE-FOR1, CRZR-RACE-FOR2, and CRZR-RACE-FOR3) and two plasmid-specific reverse primers (pDNR-LIB 3 -88 REV and pDNR-LIB 3 -25 REV) (Supplementary Table 1). The PCR product of each reaction was purified and used for the subsequent PCR reaction. The final RACE product was cloned and sequenced as described earlier.

Using the *R. prolixus* genome (Mesquita et al., 2015), a gene-specific primer was designed to obtain the full 5′ end of the Rhopr-CRZR cDNA sequence. To confirm the sequences obtained from the PCR reactions, CRZR-FOR0, and CRZR-REV4 (Supplementary Table 1) were used to amplify the largest Rhopr-CRZR cDNA fragment. The resulting PCR product was also cloned and sequenced as described earlier. Multiple independent clones were sequenced to ensure base accuracy.

### Rhopr-CRZR sequence analysis

The seven transmembrane domains of the Rhopr-CRZR were predicted using TMHMM Server v. 2.0 (http://www.cbs.dtu.dk/services/TMHMM/). The exon-intron boundaries were predicted using BLAST and confirmed with a splice site prediction software (http://www.fruitfly.org/seq_tools/splice.html). The N-linked glycosylation sites and intracellular phosphorylation sites were predicted using NetGlyc 1.0 Server (http://www.cbs.dtu.dk/services/NetNGlyc/) and NetPhos 2.0 Server (http://www.cbs.dtu.dk/services/NetPhos/), respectively. The RhopCRZR amino acid sequence was aligned with the sequences from the following cloned CRZRs: *D. melanogaster* (JC7896), *M. sexta* (AAR14318.1), *A. gambiae* (AY301275), *M. domestica* (AEI91710.1), and *B. mori* (NP-001127719.1) using MAFFT (http://mafft.cbrc.jp/alignment/server/index.html).

### Preparation of expression vector and receptor functional assay

The ORF of the two Rhopr-CRZR variants were amplified and a Kozak translation initiation sequence was introduced at the 5′ end using primers listed in Supplementary Table 2. The resulting product was cloned into pGEM-T Easy vector and subcloned into pIRES2-ZsGreen1 (Clontech, Mountain View, CA, USA).

Chinese hamster ovary (CHO) cells stably expressing aequorin (CHOK1-aeq) (Paluzzi et al., 2010) were cultured in complete media (94% Dulbecco's Modified Eagle Medium Nutrient Mixture F12-Ham (DMEM/F12) (Life Technologies Corporation, Carlsbad, CA, USA), 5% heat-inactivated fetal bovine serum (FBS), and 1% penicillin/streptomycin) containing 200 μg/mL Geneticin. The cells were incubated at 37°C in 5% CO_2_, and then transiently transfected with the expression vector for the receptor transcript variants using X-tremeGENE 9 DNA transfection reagent (Roche Applied Science, Indianapolis, IN, USA) at a ratio of 2:1 (transfection reagent to expression vector) using the manufacturer recommended protocol. Cells were also transiently transfected with an empty expression vector as a negative control.

The bioluminescence assay was performed 72 h post-transfection. The cells were first harvested with PBS/EDTA solution and suspended in bovine serum albumin BSA media (DMEM/F12 containing 1% BSA and 1% penicillin/streptomycin). The cells were incubated in the dark for 4–5 h at room temperature with 5 μM (final concentration) coelenterazine h (Promega, Madison, WI, USA) and then diluted 10-fold using BSA media. Various peptide concentrations were prepared with BSA media and loaded in triplicates on an opaque 96-well microplate. Cells were loaded into each well using an automated injector unit and luminescence was recorded over 15 s using a Wallac Victor2 plate reader (Perkin Elmer, San Diego, CA, USA). The peptides used were Rhopr-CRZ (pQTFQYSRGWTNamide), Rhopr-AKH (pQLTFSTDWamide), and Rhopr-ACP (pQVTFSRDWNAamide). Wells containing only the BSA media served as a blank control while wells containing 25, 50, and 100 μM ATP served as positive controls. Dose-response curves were obtained by averaging three replica plates and the EC_50_ values were determined using Prism5 software.

### Spatial expression pattern of Rhopr-CRZR using quantitative PCR (qPCR)

Rhopr-CRZR transcript expression was examined in various tissues from 5th instar *R. prolixus*. Tissues were dissected from 4 to 6 weeks post-fed (as previous instar) insects in nuclease-free phosphate-buffered saline (PBS) (Sigma–Aldrich, Oakville, ON, Canada). To determine the expression of the Rhopr-CRZR transcript around ecdysis, the CNS was dissected from 4th instar *R. prolixus* 3 days before ecdysis (DBE), 2 DBE, 1 DBE, 2 h post-ecdysis (2 HPE) into 5th instar, 4 HPE, 1 day post-ecdysis (DPE), 3 DPE, and 6 DPE.

Total RNA was extracted from each tissue using PureLink® RNA Mini Kit (Life Technologies Corporation, Carlsbad, CA, USA) which was then used for cDNA synthesis with iScript™ Reverse Transcription Supermix for RT-qPCR (Bio-Rad Laboratories Ltd., Mississauga, ON, Canada). The cDNA was diluted 10-fold and used as a template for the qPCR reaction. The Rhopr-CRZR gene and the reference genes (alpha-tubulin, beta-actin, and ribosomal protein 49) were amplified using primers designed over exon/exon boundaries (Supplementary Table 3; Paluzzi et al., 2010; Paluzzi and O'Donnell, 2012). The qPCR reactions were carried out on the CFX384 Touch™ Real-Time PCR Detection System (Bio-Rad, Mississauga, ON, Canada) using SsoFAST™ EvaGreen® Supermix (Bio-Rad Laboratories Ltd., Mississauga, ON, Canada). The amplification conditions were as follows: initial denaturation at 95°C for 30 s, 40 cycles of denaturation at 95°C for 5 s, annealing at 60°C for 5 s, and extension at 72°C for 5 s. Three biological replicates with three technical replicates were performed including a no-template control. The melting curve analysis was performed and the qPCR products were run on a gel and cloned to confirm that the specific transcript was amplified. The relative expression levels were determined using the ΔΔCt method and the fold differences were normalized to the three reference genes using the geometric averaging of the transcript levels.

### Fluorescent *in situ* hybridization

The distribution of cells expressing the Rhopr-CRZR mRNA within the brain was determined using fluorescent *in situ* hybridization (FISH). To synthesize the sense and antisense probes, Rhopr-CRZR was amplified via PCR using CNS cDNA as a template. T7 promoter sequence at the 5′end of the sense strand and the 3′ end of the antisense strand was added to the PCR products using the primers listed in Supplementary Table 4. The PCR amplification conditions were as follows: initial denaturation at 94°C for 3 min, followed by seven cycles of 94°C for 30 s, 58°C for 30 s and 72°C for 90 s, 30 cycles of 94°C for 30 s, 62°C for 30 s and 72°C for 90 s, and a final extension at 72°C for 10 min. The PCR products were purified and were labeled using the DIG RNA Labeling Kit (Roche Applied Science, Mannheim, Germany) following the manufacturer's protocol and stored at −20°C until use.

The brains were dissected in nuclease-free phosphate-buffered saline (PBS) and fixed in 4% paraformaldehyde prepared in PBST (PBS containing 0.1% Tween) for 1 h at room temperature. FISH was performed as previously described (Defferrari et al., 2016) using 150 ng of the anti-sense, or sense for controls, DIG-labeled Rhopr-CRZR RNA probes. No staining was observed in the controls. The cell-specific spatial expression of the transcript was viewed under a Zeiss Laser scanning confocal microscope LSM510 and LSM image browser software (Carl Zeiss, Jena, Germany).

### Double stranded RNA synthesis and delivery

The RNA probes were synthesized in a similar manner to the FISH probes. Rhopr-CRZR was used as a template for the experimental dsRNA whereas pGEM-T East vector (Promega, Madison, WI, USA) was used as a template for the control, ampicillin resistance gene (ARG). The dsRNA was synthesized and purified using the T7 Ribomax Express RNAi System (Promega, Madison, WI, USA), following the manufacturer-supplied protocol.

Adult *R. prolixus* were anesthetized briefly with CO_2_ and injected with 2 μL of 2 μg/μL of dsCRZR or dsARG into the thorax using a 5 μL Hamilton syringe. Insects were left for 30 min at room temperature to recover and then kept in an incubator at 28°C on a 16 h: 8 h light/dark cycle. To determine knockdown efficiency, qPCR was performed 3 days post-injection.

### Heartbeat assay: *in vivo*

The heartbeat of adult *R. prolixus* injected with either dsCRZR or dsARG was measured 3 days after injection. Animals were immobilized on a Dental wax-coated dissecting dish ventral-side down. Their wings were cut to expose the transparent dorsal cuticle and the *in vivo* heartbeat was counted by eye per minute under the microscope.

### Heartbeat assay: *in vitro*

Adult *R. prolixus* injected 3 days earlier with either dsCRZR or dsARG were dissected from the ventral surface under physiological saline (150 mM NaCl, 8.6 mM KCl, 2 mM CaCl_2_, 4 mM NaHCO_3_, and 8.5 mM MgCl_2_, 5 mM HEPES, pH 7.0). The digestive and reproductive systems were removed to expose the dorsal vessel. Electrodes attached to an impedance converter (UFI model 2991, Morro Bay, CA, USA) were positioned on either side of the dorsal vessel between the sixth and fifth abdominal segments. The heartbeat frequency was left to stabilize in 50 μL saline and the frequency of heartbeat measured from the traces recorded on a linear Flat-bed single channel chart recorder. The saline was then exchanged for 50 μL of 10^−8^ M CRZ and the heartbeat frequency was again measured. The response to 10^−8^ M CRZ was quantified by measuring the increase in heartbeat frequency compared to saline control.

### Effect of Rhopr-CRZR on ecdysis and cuticle coloration

Fourth instar *R. prolixus* were injected with 1 μL of 2 μg/μL dsARG or dsCRZR 10 days post feeding, and kept in an incubator at 28°C on a 16 h: 8 h light/dark cycle. The timing of ecdysis and the coloration of the cuticle post-ecdysis were monitored daily.

## Results

### Rhopr-CRZR analysis

**Figure 1:**
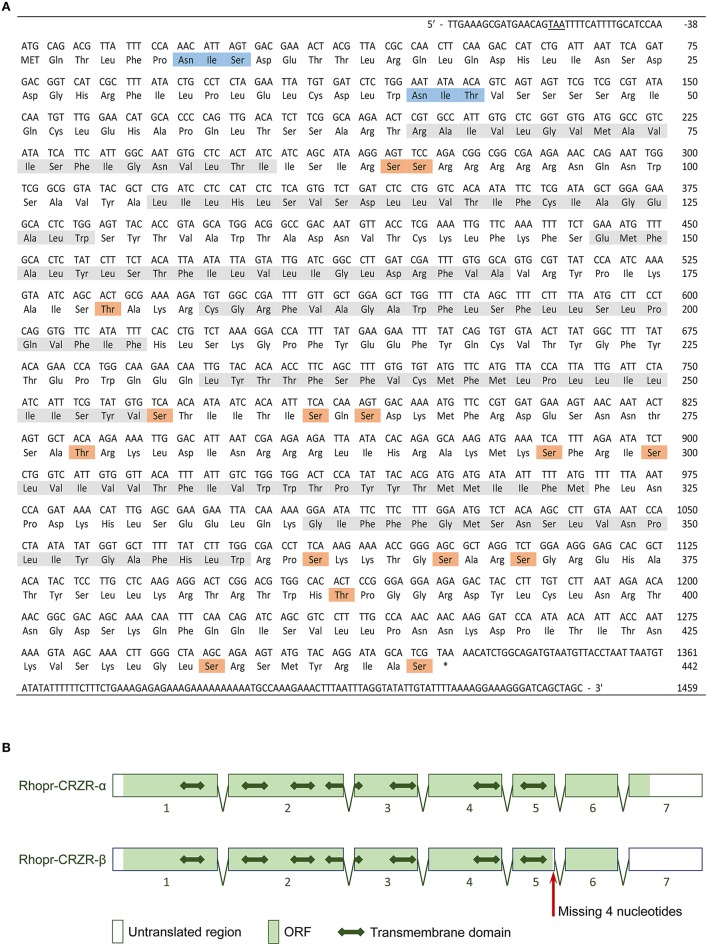
**Rhopr-CRZR-α sequence and gene structure**. **(A)** The cDNA sequence and its deduced amino acid sequence of Rhopr-CRZR-α. The numbering of the amino acids starts with the first methionine of the ORF. The stop codon upstream of the start codon is underlined. The seven transmembrane helices, the predicted N-linked glycosylation sites and predicted phosphorylation sites are shaded in gray, blue and orange, respectively. **(B)** The splicing pattern of Rhopr-CRZR-α and -β as predicted using BLAST and splice site prediction software. The boxes represent exons, and the two-headed arrow represents the transmembrane domains.

**Figure 2:**
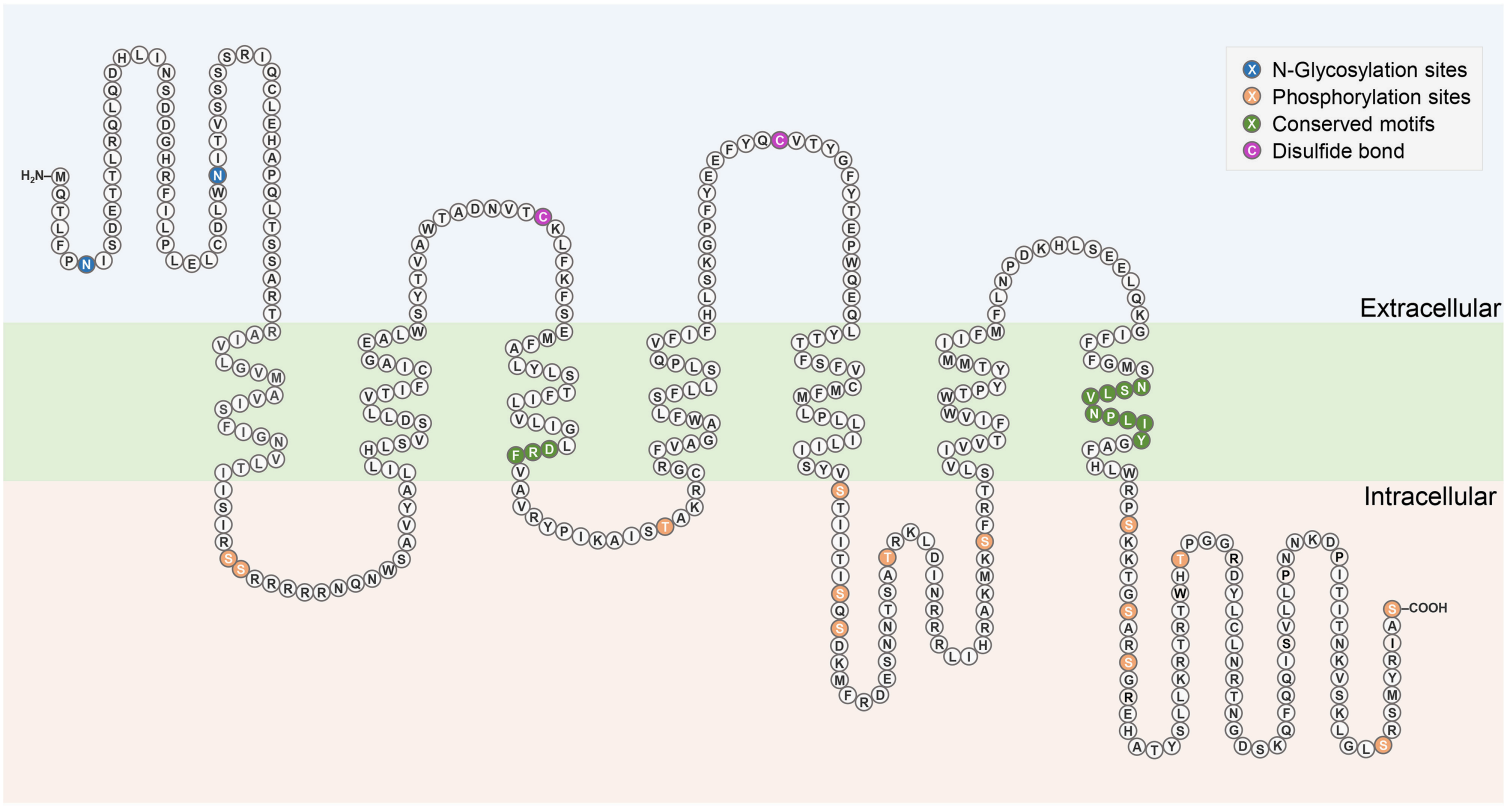
**Schematic diagram of Rhopr-CRZR-α**. The seven transmembrane domains are within the cell membrane which is shaded in green. The predicted N-glycosylation sites and phosphorylation sites are highlighted in blue and orange, respectively. The pair of cysteine residues that form a disulfide bond are highlighted in purple. Conserved motifs among Rhodopsin-like receptors are highlighted in dark green.

The complete cDNA sequence encoding Rhopr-CRZR was isolated, revealing two splice variants (Rhopr-CRZR-α and β). These encode receptors that are 441 amino acids (Rhopr-CRZR-α, accession number: KU052880) (Figure 1A) and 420 amino acids (Rhopr-CRZR-β, accession number: KU052881) (Supplementary Figure 1) in length. Rhopr-CRZR-α has seven exons and six introns. Rhopr-CRZR-β is missing four nucleotides at the end of exon five, resulting in a frame-shift, causing a truncated ORF (Figure 1B). The CRZR is predicted to have seven alpha-helical transmembrane segments in the ORF as well as three intra- and three extra-cellular loops (Figure 2). The N-terminal domain is on the extracellular side and the C-terminal domain is on the intracellular side. Both transcripts have two putative N-linked glycosylation sites in their N-terminus as well as predicted phosphorylation sites in their intracellular domains. Moreover, two cysteine residues on the first and second extracellular loops are present (Figure 2).

Sequence analysis of Rhopr-CRZR revealed characteristics of a rhodopsin-like GPCR. A divergent Asp-Arg-Phe (DRF) amino acid sequence instead of the Asp-Arg-Tyr (DRY) motif is found at the cytoplasmic end of the third transmembrane domain (Figure 2). Another characteristic of a rhodopsin-like GPCR is the presence of an NSxxNPxxY motif in the seventh transmembrane domain which is also found in the Rhopr-CRZR (Figure 2). The multiple sequence alignment of Rhopr-CRZR with other cloned insect CRZRs shows that the protein is highly conserved, mainly over the region comprising the seven transmembrane domains and the conserved regions specific for rhodopsin-like GPCRs (Supplementary Figure 2).

### Functional characterization of Rhopr-CRZR

**Figure 3:**
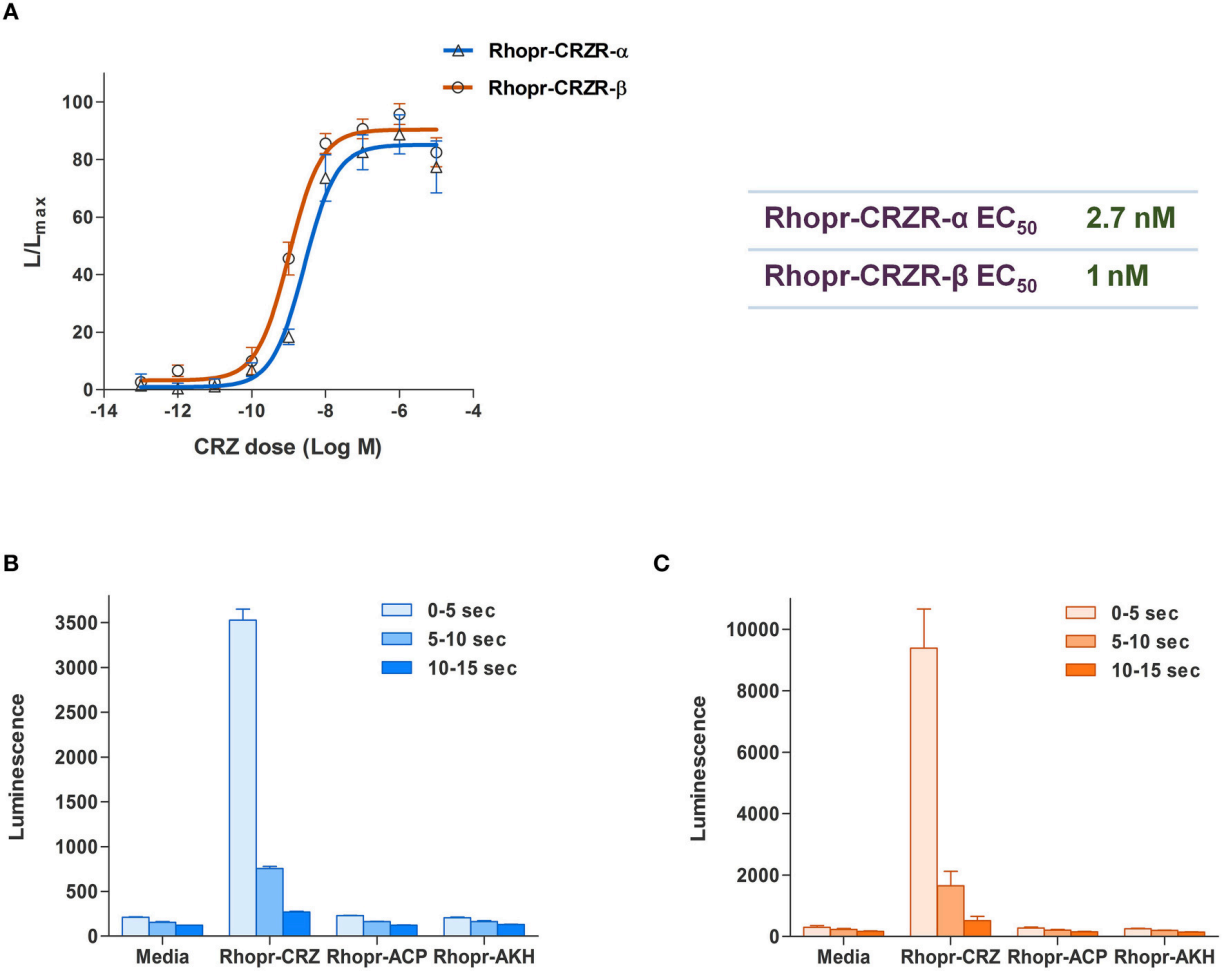
**Functional receptor assay of Rhopr-CRZR-α and β transiently expressed in CHOK1-aeq cells**. **(A)** Dose-response curve of the luminescence response following the addition of Rhopr-CRZ to cells expressing Rhopr-CRZR-α or β. The EC_50_ values of Rhopr-CRZR-α and β are 2.7 and 1 nM, respectively. Kinetics of the bioluminescence response of Rhopr-CRZR-α **(B)** and Rhopr-CRZR-β **(C)** recorded every 5 s over a 15 s interval following the addition of media, Rhopr-CRZ, Rhopr-ACP, and Rhopr-AKH at a dose of 10^−6^ M. Rhopr-ACP and Rhopr-AKH were without effect on these receptors. Values represent mean ± error of mean (*n* = 3).

To determine specificity of the cloned Rhopr-CRZR to its putative ligand, Rhopr-CRZ, a calcium mobilizing assay was used where both receptor transcripts were separately transiently transfected into CHOKI-aeq cells. Rhopr-CRZR-α and β were dose-dependently activated by Rhopr-CRZ with an EC_50_ value of 2.7 and 1 nM, respectively (Figure 3A). Maximum luminescence was seen during the first 5 s following activation of either receptor with Rhopr-CRZ (Figures 3B,C). Neither receptor was activated by the evolutionarily-related peptides, Rhopr-AKH, or Rhopr-ACP (Figures 3B,C), which contain some sequence similarity (Table 1). Moreover, control cells transfected with an empty vector did not elicit a response.

**Table 1:** **Amino acid sequences and similarities between Rhopr-CRZ, Rhopr-ACP, and Rhopr-AKH in ***R. prolixus*****.

**Neuropeptide**	**Amino acid sequence**												
Rhopr-CRZ	pQ	–	T	F	Q	Y	S	R	G	W	T	N	amide
Rhopr-ACP	pQ	V	T	F	–	–	S	R	D	W	N	A	amide
Rhopr-AKH	pQ	L	T	F	–	–	S	T	D	W	–	–	amide

### Transcript expression profile of Rhopr-CRZR

**Figure 4:**
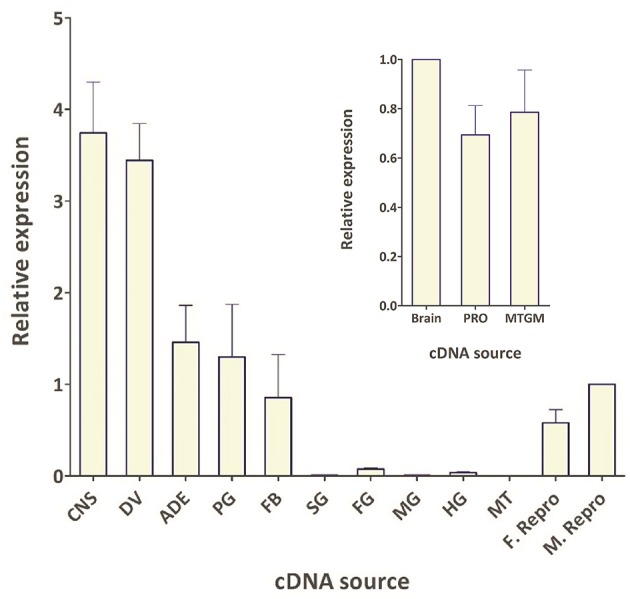
**Spatial expression pattern of Rhopr-CRZR transcript in 5th instar ***R. prolixus*** using qPCR**. The expression was analyzed in the central nervous system (CNS), dorsal vessel (DV), abdominal dorsal epidermis (ADE), prothoracic glands and its associated fat body (PG), fat body (FB), salivary glands (SG), foregut (FG), midgut (MG), hindgut (HG), Malpighian tubules (MT), female reproductive system (F. Repro), and male reproductive system (M. Repro). Fold difference is relative to transcript levels in M. Repro cDNA. Inset shows the expression in the brain plus suboesophaegeal ganglion (Brain), prothoracic ganglion (PRO), and mesothoracic ganglionic mass (MTGM). Fold difference is relative to transcript levels in the brain cDNA. The qPCR was performed on three biological replicates with three technical replicates each. Values represent mean ± error of mean.

**Figure 5:**
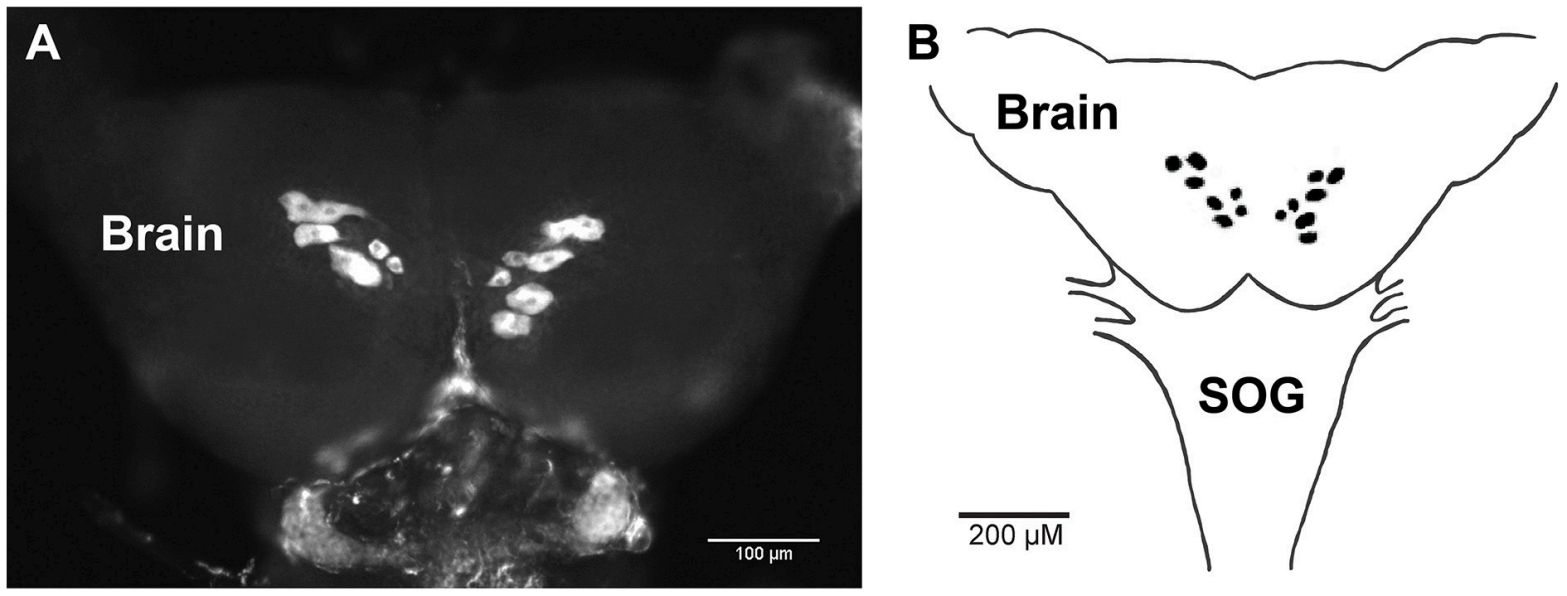
**Fluorescent ***in situ*** hybridization of Rhopr-CRZR transcript in the brain of 5th instar ***R. prolixus*****. **(A)** Confocal microscopy image of the brain revealing a group of seven bilaterally paired medial cells on the dorsal surface. **(B)** Diagram illustrating the location of cells in the brain containing the Rhopr-CRZR transcript. The fluorescent *in situ* hybridization was performed three times with eight CNSs each time. SOG, suboesophageal ganglion.

The spatial expression pattern of the Rhopr-CRZR transcript was used to determine which peripheral tissues express the receptor to give clues on the functional roles for the Rhopr-CRZ signaling pathway. In 5th instar *R. prolixus*, the highest transcript expression was found in the CNS (Figure 4). Similar expression levels were found in the different parts of the CNS; the brain and suboesophageal ganglion (Brain), the prothoracic ganglion (PRO), and mesothoracic ganglionic mass (MTGM) (Figure 4 inset). Cells that express the Rhopr-CRZR mRNA were localized in the brain using fluorescent *in situ* hybridization (FISH) (Figure 5). On the dorsal side of the brain, a group of 7 bilaterally paired medial cells were observed, two of which are smaller in diameter than the other 5. On the basis of size, shape and location, these appear to be a subset of the medial neurosecretory cells.

The spatial expression pattern also revealed high expression in the dorsal vessel, similar to that of the CNS (Figure 4). The receptor transcript was also expressed at moderate levels in the abdominal dorsal epidermis, and prothoracic glands with associated fat body. There was minimal transcript expression in the foregut, midgut, and hindgut. Interestingly, transcript expression was also found in the female and male reproductive tissues (Figure 4).

### Rhopr-CRZR role in regulating heartbeat rate

**Figure 6:**
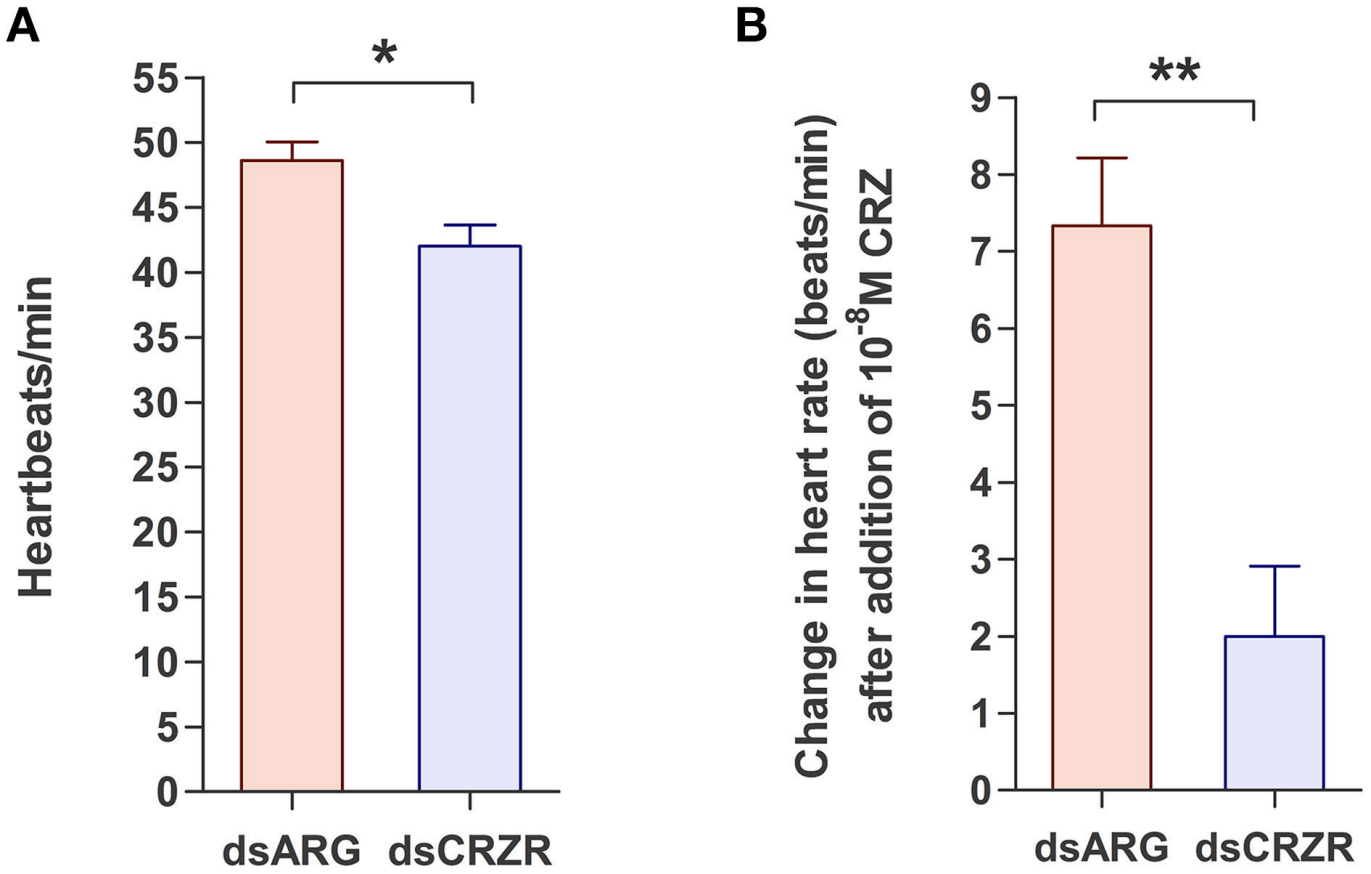
**The effect of Rhopr-CRZR on heartbeat in adult ***R. prolixus***. 2 μg of dsARG or dsCRZR was injected into adult ***R. prolixus*** and the effect on heartbeat was measured 3 days post injection**. Values represent mean ± error of mean (*n* = 5). **(A)** Heartbeat frequency *in vivo* in dsARG and dsCRZR injected insects. There is a significant decrease in heartbeats per minute in dsCRZR injected insects (unpaired *t*-test, ^*^p = 0.0188). **(B)** The effect of 10^−8^ M Rhopr-CRZ *in vitro* on heartbeat rate in dsARG and dsCRZR injected insects. Rhopr-CRZ at 10^−8^ M significantly increased the heartbeat rate in dsARG injected insects but not in dsCRZR injected insects (unpaired *t*-test, ^**^p = 0.0096).

The percent knockdown of Rhopr-CRZR transcript after injection of dsCRZR into adult *R. prolixus* was quantified by qPCR in the dorsal vessel. It was found to be knocked down by 86.9 ± 2.9% relative to control dsARG injected insects 3 days after injection. The physiological effects of this decrease in Rhopr-CRZR on heartbeat frequency was studied *in vivo* and *in vitro* in adult *R. prolixus*. *In vivo*, the heartbeat frequency of insects previously injected with dsCRZR (42 ± 1.63 beats/min, n = 5) was decreased by 13.6% compared to control insects injected with dsARG (48.6 ± 1.4 beats/min, *n* = 5) (unpaired *t*-test, *p* = 0.0188) (Figure 6A). Previously, Rhopr-CRZ has been shown to increase heartbeat frequency in a dose-dependent manner *in vitro* in 5th instar *R. prolixus* (Patel et al., 2014). To verify the *in vivo* results, the heartbeat frequency was investigated *in vitro* to determine if the reduced heartbeat frequency *in vivo* was due to the decrease in Rhopr-CRZR. After the addition of 10^−8^ M Rhopr-CRZ, the heartbeat frequency of dsARG injected insects increased by 7.33 ± 0.8 beats/min (*n* = 5) whereas the heartbeat frequency only increased by 2 ± 0.9 beats/min (*n* = 5) in dsCRZR injected insects (unpaired *t*-test, *p* = 0.0096) (Figure 6B).

### The possible role of Rhopr-CRZ in regulating ecdysis

**Figure 7:**
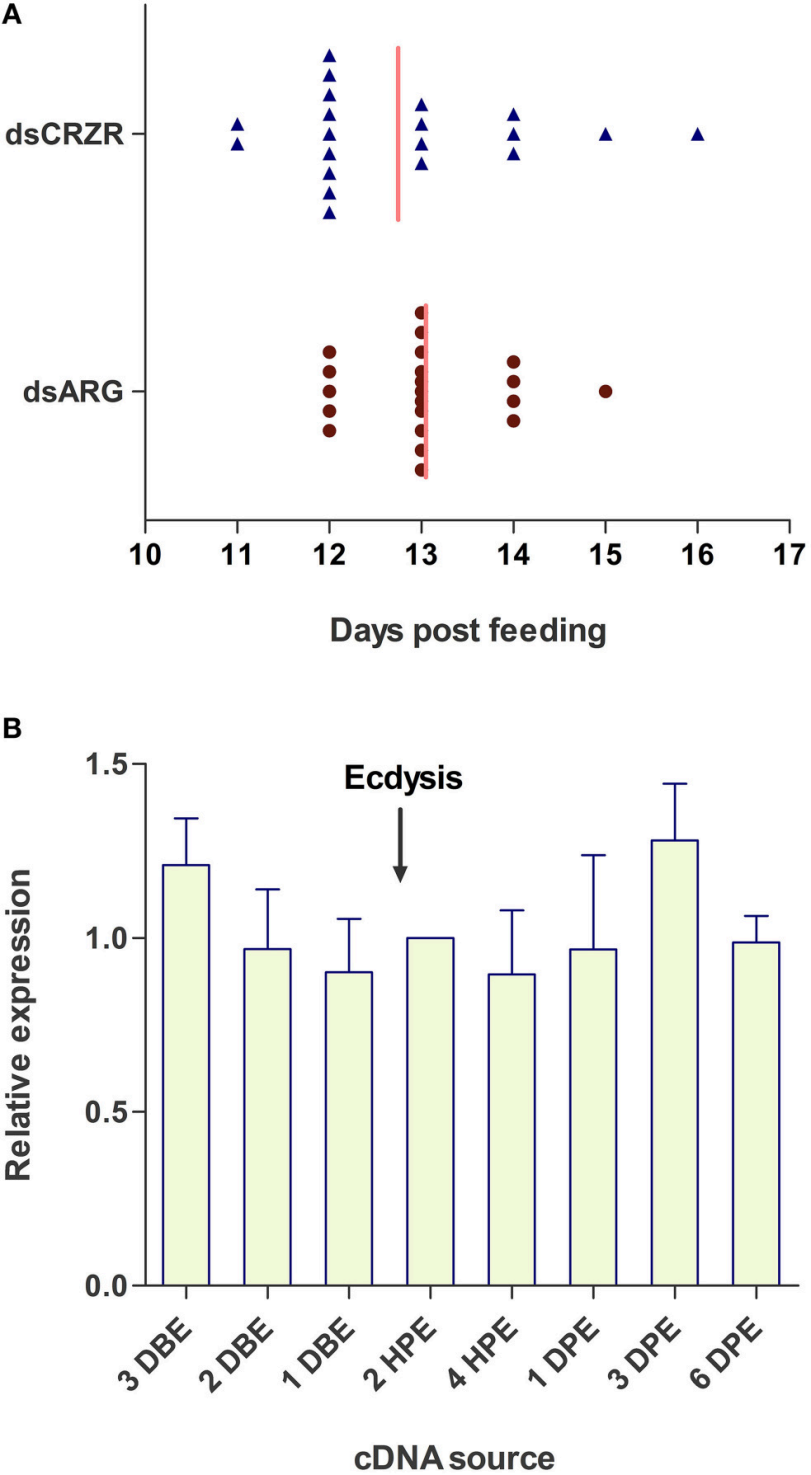
**The effect of Rhopr-CRZR on ecdysis in ***R. prolixus*****. **(A)** 4th instar *R. prolixus* were injected with 2 μg of dsARG or dsCRZR 10 days post feeding. Ecdysis took place 11–16 days post-feeding. There was no significant effect on the timing of ecdysis between dsARG RNA and dsCRZR injected insects; however, dsCRZR injected insects were observed to undergo ecdysis a day earlier causing a shift in the median (vertical line). **(B)** Temporal expression analysis of Rhopr-CRZR transcript in the CNS by qPCR indicated that the receptor expression is not up-regulated around the timing of ecdysis. The expression was analyzed in 4th instar CNS 3 days before ecdysis (DBE), 2 DBE, 1 DBE, 2 h post-ecdysis (HPE) into 5th instar, 4 HPE, 1 day post-ecdysis (DPE), 3 DPE, and 6 DPE. Fold difference is relative to transcript levels of 2 HPE. The experiment was repeated on three biological replicates with three technical replicates each. Values represent mean ± error of mean.

The possible involvement of the Rhopr-CRZ-signaling pathway on ecdysis and cuticle coloration was monitored in 4th instar to 5th instar *R. prolixus*. Under the specific experimental conditions, ecdysis took place 11–16 days post-feeding of 4th instars on rabbit's blood (Figure 7A). Insects injected with dsARG (*n* = 21) and dsCRZR (*n* = 20) displayed no significant difference in ecdysis behavior or timing of ecdysis (Figure 7A); however, the peak of ecdysis for the dsARG injected insects was 13 days post-feeding compared to 12 days post-feeding for dsCRZR injected insects. Perhaps this might be caused by decreasing heartbeat frequency and influencing hemolymph circulation. The percent knockdown of the Rhopr-CRZR transcript in the CNS was 79 ± 1% relative to dsARG injected bugs 3 days after injections. The transcript levels of Rhopr-CRZR at different time points around ecdysis were measured using qPCR. There was no significant difference in the expression of Rhopr-CRZR from 3 days pre-ecdysis to 3 days post-ecdysis (Figure 7B).

### The possible role of Rhopr-CRZR in regulating cuticle coloration

**Figure 8:**
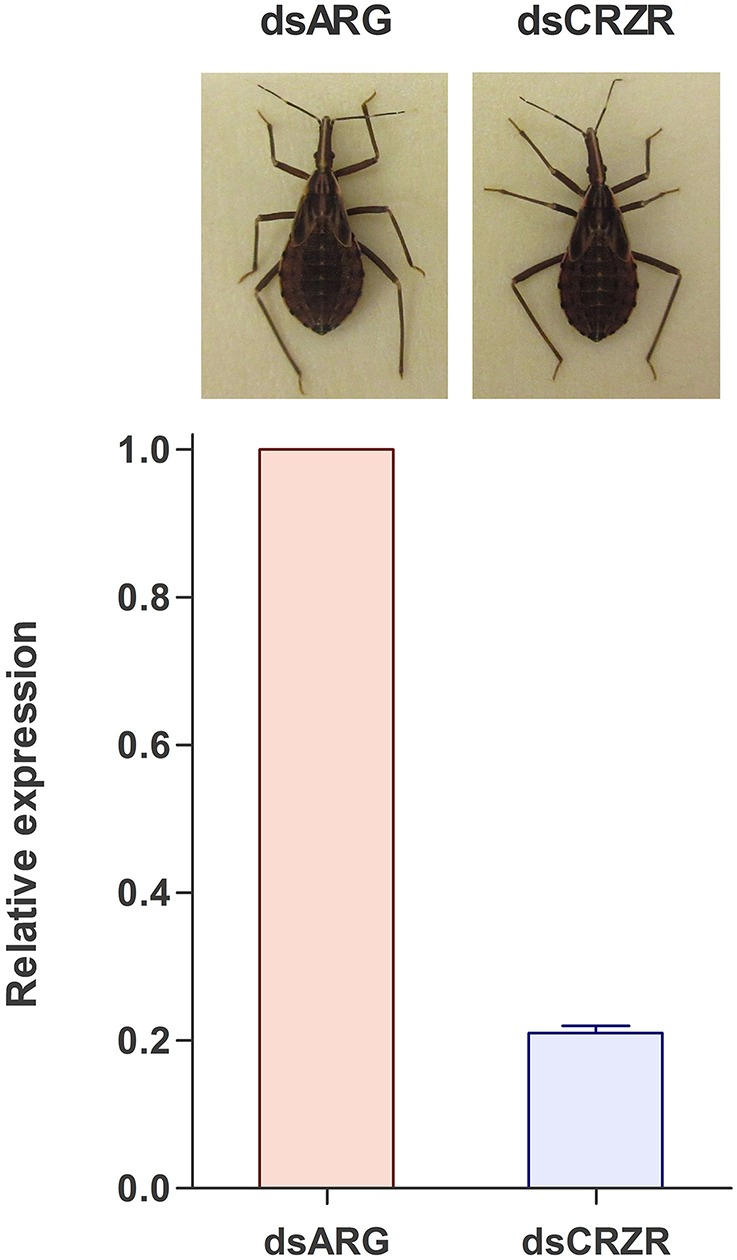
**The effect of decreasing the Rhopr-CRZR transcript levels on cuticle coloration**. Fourth instar *R. prolixus* were injected with 2 μg of dsARG or dsCRZR, decreasing the Rhopr-CRZR transcript by 79 ± 1%. Following ecdysis to 5th instar, there was no difference between the dsARG- and dsCRZR-injected insects. The final cuticle color of the insects was the same in both cases.

Following ecdysis, the coloration of the cuticle was monitored to determine the effect of decreases in Rhopr-CRZR levels on cuticle coloration. Insects injected with dsARG (*n* = 21) and dsCRZR (*n* = 20) did not show any difference in coloration or the time it takes to darken the cuticle (Figure 8).

## Discussion

In this study, we isolated and characterized the cDNA sequence encoding Rhopr-CRZR. The CRZR is present in a number of insects such as *A. gambiae, B. mori, D. melanogaster, M. sexta*, and *M. domestica*. Their sequences are highly conserved and similar to the CRZR sequence isolated and cloned in *R. prolixus*. Two CRZRs have been found in *R. prolixus*, whereas only one CRZR has been identified in other insects. The two receptors are a result of alternative splicing causing one receptor (Rhopr-CRZR-α) to have a larger C-terminal than the other (Rhopr-CRZR-β). Alternative splicing causes structural diversity where a single transcript can generate more than one mRNA (Journot et al., 1994). This phenomenon has been selected in evolution as it can regulate or modify the intensity and specificity of the binding of proteins to the receptor (Journot et al., 1994).

Specificity of the Rhopr-CRZR was determined using CHOK1-aeq cells where only Rhopr-CRZ elicited a response, confirming ligand binding. Through gene duplication, and successive specialization, the CRZ, AKH, and ACP-signaling pathways emerged (Hauser and Grimmelikhuijzen, 2014). To deduce the specificity of the Rhopr-CRZR to Rhopr-CRZ, Rhopr-AKH, and Rhopr-ACP were tested to examine if they elicited a response in the functional receptor assay, and they did not. Despite the sequence similarity between these neuropeptides, there is no cross-reactivity between the different signaling pathways (Hansen et al., 2010; Patel et al., 2014).

As a preliminary investigation into possible roles of Rhopr-CRZ in the CNS we performed *in situ* hybridization on the brain of 5th instars. Interestingly, seven bilaterally-paired median cells express the receptor transcript. These cells are of a size and location of medial neurosecretory cells, and so Rhopr-CRZ may be involved in coordinating neuroendocrine activities in *R. prolixus*. Similar CRZR transcript expression in a subset of medial neurosecretory cells has also been shown in *Drosophila* (Zandawala personal communication). Clearly, additional *in situ* hybridization studies will be required for all tissues over a developmental cycle to gain further insight into the CRZ-signaling pathways.

Quantitative analysis of the Rhopr-CRZR transcript expression can also reveal the peripheral tissues that express the receptor, giving clues for functional roles. The presence of the receptor implies that CRZ should influence the activity of that tissue. Rhopr-CRZR transcript expression was seen throughout the CNS and associated with peripheral tissues. These included the dorsal vessel, abdominal dorsal epidermis, prothoracic glands and associated fat body, abdominal fat body, and male and female reproductive tissue.

High expression of Rhopr-CRZR transcript in the dorsal vessel was not unexpected since Rhopr-CRZ stimulates heartbeat frequency in *R. prolixus* (Patel et al., 2014). To further confirm the role of the CRZ-signaling pathway on the dorsal vessel in *R. prolixus*, the Rhopr-CRZR transcript was knocked down using dsRNA. Significantly lowering the transcript levels in adults led to a decrease in heartbeat frequency *in vivo* indicating that CRZ is involved in the regulation of heartbeat in the intact animal. Moreover, Rhopr-CRZR knockdown significantly reduced the effect of Rhopr-CRZ on heartbeat frequency *in vitro*. Thus, Rhopr-CRZ was less effective at increasing heartbeat rate *in vitro* in insects which had knockdown of the Rhopr-CRZR transcript. It has been previously shown in *R. prolixus* that CRZ increases the heartbeat frequency in a dose-dependent manner (Patel et al., 2014). This is the first report to show that the CRZ-signaling pathway is involved in the regulation of heartbeat frequency in intact adult *R. prolixus*.

The expression of the Rhopr-CRZR in the prothoracic glands implies that the CRZ-signaling pathway may play a role in the molt cycle since the prothoracic glands is essential for releasing ecdysteroids that regulate molting. Wigglesworth (1934) found that following a blood meal, a hormone which was later termed the prothoracictropic hormone (PTTH), acted on the prothoracic glands, triggering the secretion of ecdysteroids into the hemolymph (Kataoka et al., 1991). These ecdysteroids coordinate molting by regulating the expression of specific neuropeptide genes coding for, for example, PETH and ETH (Žitňan et al., 1996, 1999). To determine if Rhopr-CRZ also initiates ecdysis in *R. prolixus*, similar to that found in *M. sexta*, the Rhopr-CRZR was knocked down prior to ecdysis; however, data showed that it did not have an effect on timing or duration of ecdysis under the experimental protocol used here. Moreover, receptor transcript levels were not upregulated in the CNS around the time of ecdysis, again indicating that the Rhopr-CRZR transcript levels were not influenced during the timing of ecdysis or behaviors associated with ecdysis. The CRZ-signaling pathway may not be involved in ecdysis, but perhaps involved earlier in the molting process. For example, it could play a role with PTTH in regulating production and release of ecdysteroids, implying that PTTH may not act alone and possibly the ingestion of a large blood meal may also trigger the release of CRZ to act on the prothoracic glands, also triggering the release of ecdysteroids.

Expression of Rhopr-CRZR transcript was observed in the abdominal dorsal epidermis suggesting the involvement of the corazonin-signaling pathway in cuticle coloration as found in locusts (Tawfik et al., 1999). In *R. prolixus*, following ecdysis, the newly molted insect has a soft, and light colored cuticle that hardens and darkens in the subsequent hours post-ecdysis. The effect of Rhopr-CRZ on the coloration of the cuticle was examined by decreasing the RhoprCRZR transcript levels using RNAi. Following ecdysis, both the control and experimental insects were the same color and darkened at the same rate resulting in identical cuticle colors. Therefore, despite a significant knockdown of the transcript, there was no effect on coloration. Even though the CRZ peptide was found to induce tanning of the cuticle in locusts (Tawfik et al., 1999; Tanaka, 2000), it did not have that effect on other insects such as *G. mellonella, G. bimaculatus*, and *B. mori* (Hua et al., 2000; Hansen et al., 2001), and may also not be involved in this in *R. prolixus*.

Perhaps the expression of the Rhopr-CRZR transcript in the anterior dorsal epidermis is due to its involvement in other functions such as cuticle secretion. During ecdysis, the epidermal cells elongate, separating themselves from the old cuticle. Before molting to the next instar, a new cuticle is set down by first secreting the epicuticle, the exocuticle and finally the endocuticle. The Rhopr-CRZ pathway could be involved in the regulation of this process.

It was interesting and unexpected to find expression of Rhopr-CRZR transcript associated with the reproductive system since reproductive functions for the CRZ-signaling pathway in insects have not been reported. CRZ is believed to have a vertebrate homolog, GnRH (Hauser and Grimmelikhuijzen, 2014; see Roch et al., 2014) and so the CRZ-signaling pathway may have reproduction functions similar to GnRH. GnRH is a key regulator of reproductive maturation in vertebrates and recently GnRH-type and CRZ-type signaling systems were identified in echinoderms illustrating the paralogous CRZ-signaling pathway is present in the common ancestor of the Bilateria (Semmens et al., 2016).

Despite CRZ having a conserved structure throughout evolution, it does not have a conserved function - from affecting cardiovascular activity in some insects to affecting ecdysis and cuticle coloration in others. Perhaps these functions are not distinct and fall under the common factor of regulating physiological stress (Boerjan et al., 2010). Examples of stressors that threaten homeostasis include extreme temperature, humidity, food shortage, light intensity, and excessive population density. Cardiovascular activity can be induced by a number of physiological stressors. For example, after the ingestion of a large blood meal in *R. prolixus*, the contractions of the anterior midgut push the hemolymph to the posterior end of the insect. To help circulate the hemolymph throughout the bug, four pairs of ostia or valves that make up the heart, open, taking in hemolymph which is propelled anteriorly through the dorsal vessel by heart muscle contractions (Chiang et al., 1990). CRZ may increase heartbeat frequency in *R. prolixus* in order to accelerate hemolymph circulation during times of stress (e.g., massive blood meal). Having a cardioacceleratory effect also results in Rhopr-CRZ itself being circulated. Cuticle darkening in locusts is thought to be a result of different stressors such as food deprivation (Veenstra, 2009), and the involvement of CRZ in ecdysis initiation in *M. sexta* may be due to a response to stress as ecdysis is a critical part of development that is dependent on nutrient and water balance (Zhao et al., 2010). Further research may uncover additional roles of CRZ, enhancing our understanding of the role that CRZ plays in various behaviors.

## Author contributions

ZH, Designed and performed all experiments, data analysis and written work. AL, Contributed to experimental design and revisions to manuscript. IO, Contributed to experimental design and revisions to manuscript.

## Funding

This work has been funded by the Natural Sciences and Engineering Research Council of Canada (NSERC) to AL (RGPIN 2014-06253) and IO (RGPIN 8522-12).

### Conflict of interest statement

The authors declare that the research was conducted in the absence of any commercial or financial relationships that could be construed as a potential conflict of interest.
