# Supplementary material for: Identification and Characterization of the Corazonin Receptor and Possible Physiological Roles of the Corazonin-Signaling Pathway in Rhodnius prolixus
Source: Front Neurosci. 2016 Aug 3;10:357. doi: 10.3389/fnins.2016.00357 (PMC4971055; doi:10.3389/fnins.2016.00357)
Supplement: Supplementary file 1 [file DataSheet1.DOCX]

Supplementary Material

**Identification and characterization of the corazonin receptor and possible physiological roles of the corazonin-signaling pathway in *Rhodnius prolixus.***

**Zina Hamoudi ^a,^ *, Angela B. Lange ^a^, and Ian Orchard ^a^**

^a^ Department of Biology, University of Toronto Mississauga, Mississauga, Ontario, Canada.

* Corresponding author

Tel.: + 1 905 828 5333

Fax: + 1 905 828 3792

Email address: [zina.hamoudi@mail.utoronto.ca](mailto:zina.hamoudi@gmail.com)

1. **Supplementary information**

**1.1 Supplementary Figures**

**
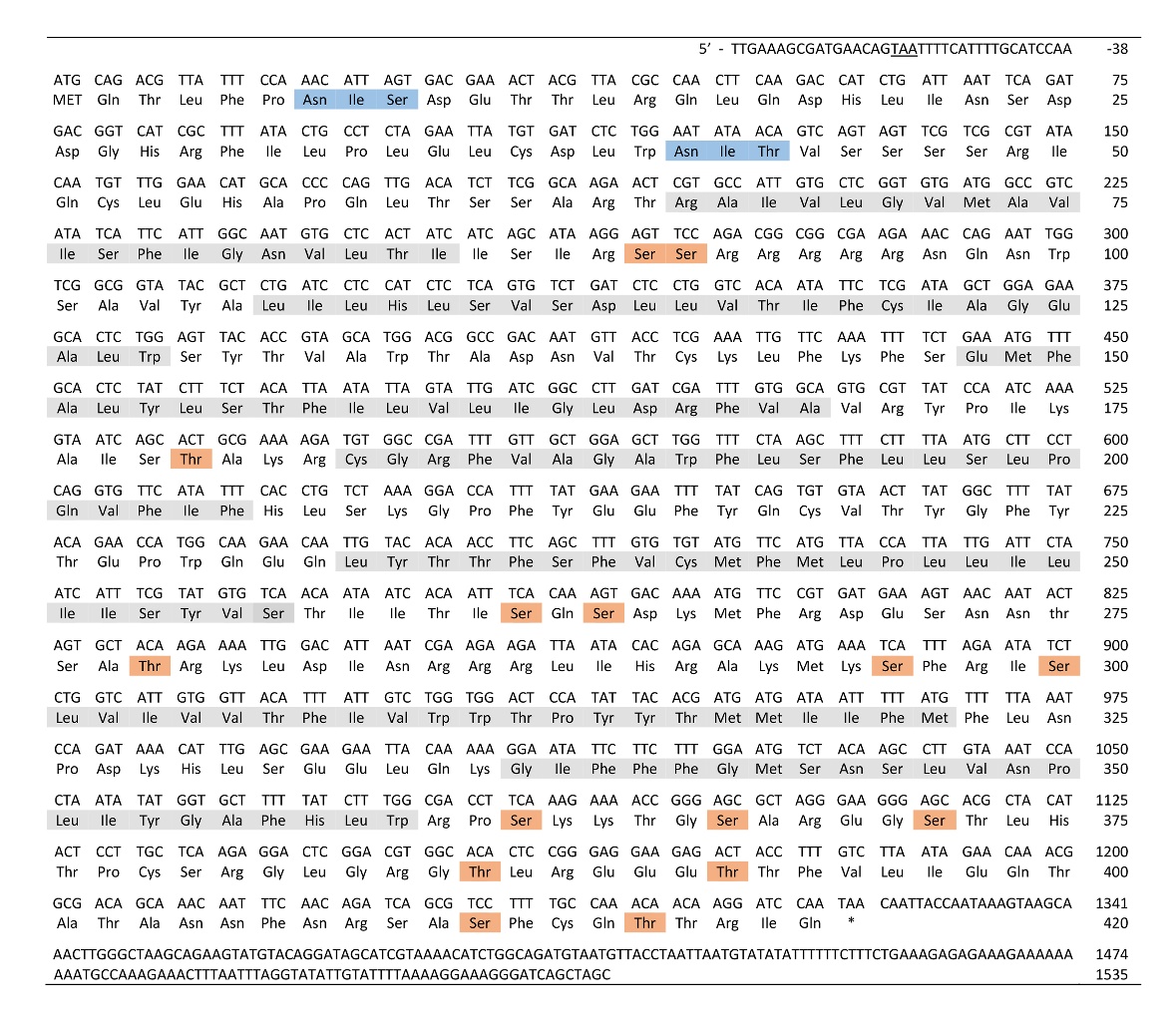
**

**Supplementary Figure 1.** The cDNA sequence and deduced amino acid sequence of Rhopr-CRZR-β. The numbering of the amino acids starts with the first methionine of the ORF. The stop codon upstream of the start codon is underlined. The seven transmembrane helices, the predicted N-linked glycosylation sites and predicted phosphorylation sites are shaded in with grey, blue and orange, respectively.


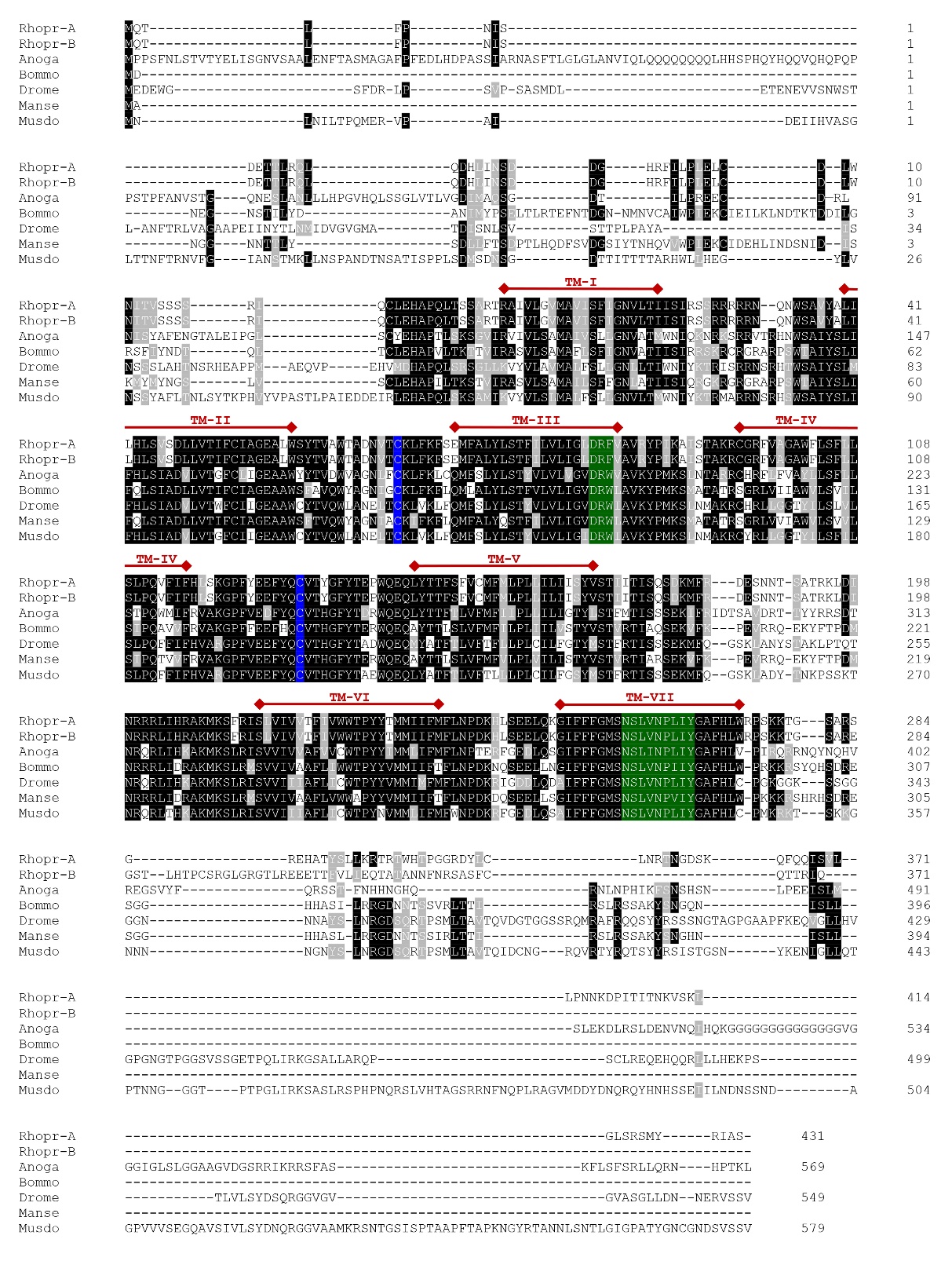


**Supplementary Figure 2.** Amino acid sequence alignment Rhopr-CRZR-α (Rhopr-α) and Rhopr-CRZR-β (Rhopr-β), *Anopheles gamb*iae (Anoga), *Bombyx mori* (Bommo), *Drosophila melanogaster* (Drome), *Manduca sexta* (Manse), and *Musca domestica* (Musdo). Identical and 50% similar amino acids across all the sequences have been highlighted in black and grey, respectively. Transmembrane helices are indicated by Tm I-VII. The conserved cysteine residues are highlighted in blue, while the conserved motifs among Rhodopsin-like receptors are highlighted in green.

**1.2 Supplementary Tables**

**Supplementary Table 1.** Primers used to clone cDNA sequence encoding Rhopr-CRZR.

| Primer | Primer Sequence (5’ to 3’) | |
| --- | --- | --- |
| *Amplify the partial sequence* | |  |
| CRZR-FOR1 | GGAATATAACAGTCAGTAGTTCGTCG |  |
| CRZR-REV1 | GATTTACAAGGCTGTTAGACATTCC |  |
| 3’ RACE | |  |
| CRZR-RACE-FOR1 | TGTGGTTACATTTATTGTCTGGTGG |  |
| CRZR-RACE-FOR2 | AAATCCAGATAAACATTTGAGCG |  |
| CRZR-RACE-FOR3 | GAATGTCTAACAGCCTTGTAAATCC |  |
| pDNR-LIB 3 -88 REV | AGTCATACCAGGATCTCCTAGGG |  |
| pDNR-LIB 3 -25 REV | GCCAAACGAATGGTCTAGAAAG |  |
| *Amplify the complete cDNA sequence* | |  |
| CRZR-FOR0 | TTGAAAGCGATGAACAG |  |
| CRZR-REV4 | GCTAGCTGATCCCTTTCC |  |

**Supplementary Table 2.** Primers used to amplify the ORF of Rhopr-CRZR-α and β and introduce the Kozak sequence.

| Primer | Primer Sequence (5’ to 3’) | |
| --- | --- | --- |
| *Rhopr-CRZR-α* | |  |
| CRZR-ORF-FOR2 | GCCACCATGCAGACGTTATTTCC |  |
| CRZR-ORF-REV1 | TTACGATGCTATCCTGTACATACTTCTG |  |
| *Rhopr-CRZR-β* | |  |
| CRZR-ORF-FOR2 | GCCACCATGCAGACGTTATTTCC |  |
| CRZR-ORF-REV2 | TTATTGGATCCTTGTTGTTTGGC |  |

**Supplementary Table 3.** Transcript specific primers used for qPCR.

| Primer | Primer Sequence (5’ to 3’) | |
| --- | --- | --- |
| *Rhopr-CRZR* | |  |
| CRZR-qPCR-FOR2 | CCGTCATATCATTCATTGGC |  |
| CRZR-qPCR-REV2 | TGGAGGATCAGAGCGTATACC |  |
| *Rhopr-beta-actin* | |  |
| Actin5c-qPCR-F | AGAGAAAAGATGACGCAGATAATGT |  |
| Actin5c-qPCR-R | ATATCCCTAACAATTTCACGTTCG |  |
| *Rhopr-alpha-tubulin* | |  |
| alphaTUB-qPCR-F | GTGTTTGTTGATTTGGAACCTACAG |  |
| alphaTUB-qPCR-R | CCGTAATCAACAGACAATCTTTCC |  |
| *Rhopr-ribosomal protein 49* | |  |
| rp49-qPCR-F | GTGAAACTCAGGAGAAATTGGC |  |
| rp49-qPCR-R | AGGACACACCATGCGCTATC |  |

**Supplementary Table 4.** Primers used to generate *in situ* probes and dsRNA for Rhopr-CRZR and ARG.

| Primer | Primer Sequence (5’ to 3’) |
| --- | --- |
| *dsRhopr-CRZR* | |
| CRZR-RNAi-FOR3 | CCTTGATCGATTTGTGGC |
| CRZR-RNAi-REV3 | AATGTAACCACAATGACCAGAG |
| CRZR-RNAi-FOR3 (with T7) | TAATACGACTCACTATAGGGAGACCTTGATCGATTTGTGGC |
| CRZR-RNAi-REV3 (with T7) | TAATACGACTCACTATAGGGAGAAATGTAACCACAATGACCAGAG |
| *dsARG* | |
| dsRNA-ARG-FOR1 | ATGAGTATTCAACATTTCCGTGTC |
| dsRNA-ARG-REV2 | AATAGTTTGCGCAACGTTG |
| dsRNA-ARG-FOR1 (with T7) | TAATACGACTCACTATAGGGAGAATGAGTATTCAACATTTCCGTGTC |
| dsRNA-ARG-REV2 (with T7) | TAATACGACTCACTATAGGGAGAAATAGTTTGCGCAACGTTG |
